# Adenine phosphoribosyltransferase deficiency and 2,8-dihydroxyadeninuria

**DOI:** 10.1007/s00467-026-07187-9

**Published:** 2026-03-13

**Authors:** Vidar O. Edvardsson, Hrafnhildur L. Runolfsdottir, Runolfur Palsson

**Affiliations:** 1 Children’s Medical Center, Landspitali University Hospital, Reykjavik, 101 Iceland; 2 Faculty of Medicine, School of Health Sciences, University of Iceland, Reykjavik, Iceland; 3 Internal Medicine Services, Landspitali University Hospital, Reykjavik, Iceland; 4 Section of Nephrology, Landspitali University Hospital, Reykjavik, Iceland

**Keywords:** Diaper stains, Crystalluria, Urinary stone disease, Crystal nephropathy, Acute kidney injury, Chronic kidney disease, Kidney failure, Pharmacotherapy

## Abstract

Adenine phosphoribosyltransferase (APRT) deficiency is a rare autosomal recessive disorder of purine metabolism causing 2,8-dihydroxyadenine urinary tract stones and crystal nephropathy. Disease manifestations include acute kidney injury (AKI), progressive chronic kidney disease (CKD), and not infrequently, kidney failure. A significant proportion of affected individuals remain asymptomatic into adulthood. The diagnosis of APRT deficiency should be considered in all children presenting with renal colic, radiolucent urinary stones and/or AKI, as well as in infants with a history of reddish-brown diaper stain. Indeed, the disorder should be considered in any person with radiolucent urinary stones and in young and middle-aged individuals with progressive CKD, particularly when a kidney biopsy reveals tubulointerstitial nephropathy of unknown cause. The presence of biallelic pathogenic *APRT* mutations identified through targeted multi-gene panels or single-gene testing confirms the diagnosis of APRT deficiency. As kidney stone analysis can be false-positive, this method can only be considered suggestive of APRT deficiency, and confirmatory testing must be carried out in all cases. Timely diagnosis and treatment with a xanthine oxidoreductase inhibitor, either allopurinol or febuxostat, have in several studies, based on different levels of evidence, been found to be both highly effective and safe in APRT deficiency. Appropriate pharmacotherapy significantly reduces kidney stone recurrence, slows CKD progression and appears to prevent the development of kidney failure. The outcome of kidney transplantation in individuals with APRT deficiency is comparable to those with other causes of kidney failure when allopurinol or febuxostat therapy is initiated before the transplant procedure.

**Graphical abstract:**

**Figure:**
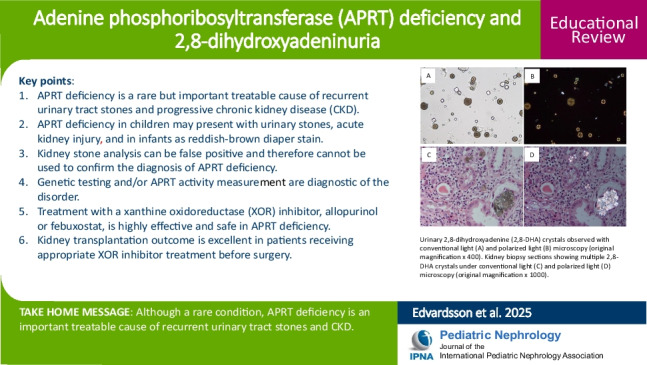
A higher resolution version of the Graphical abstract is available as Supplementary information

**Supplementary Information:**

The online version contains supplementary material available at 10.1007/s00467-026-07187-9.

## Introduction

Adenine phosphoribosyltransferase (APRT) deficiency (OMIM 102600) is a rare autosomal recessive disorder of purine metabolism characterized by the generation and urinary excretion of 2,8-dihydroxyadenine (2,8-DHA), leading to radiolucent urinary stone formation and crystal nephropathy. Affected individuals may develop acute kidney injury (AKI) episodes, progressive chronic kidney disease (CKD) and, not infrequently, kidney failure [1–3]. The diagnosis is commonly made following the incidental finding of 2,8-DHA crystalluria on urine microscopy or during screening of siblings of index cases, some of whom may be asymptomatic [1]. Partial APRT deficiency was first described in 4 asymptomatic subjects in 1968 [4], and the first individual with complete APRT deficiency was a young child with urinary tract stones reported in 1973 by Carter et al. from France [5]. The kidneys and urinary tract are the only organ systems known to be involved in APRT deficiency [3]. However, it is noteworthy that we have encountered various eye complaints in a significant proportion of our patients (personal observations) that remain to be elucidated [6]. The estimated *APRT* heterozygote frequency rate in different populations ranges from 0.4 to 1.2% [7, 8], suggesting a prevalence of the homozygous state in the range of 1:50,000–100,000 when assuming the Hardy-Weinberg equilibrium. If these data are accurate, approximately 70–80,000 cases would be expected worldwide. Despite extensive efforts, we have only been able to identify 500 cases (homozygotes and compound heterozygotes) globally by combining a search of the medical literature, data from deCODE genetics and public genomic databases, including the UK Biobank, the 100,000 Genomes Project, the Genome Aggregation Database, the Human Genetic Variation Database and the Korean Variant Archive, as well as data obtained through the Icelandic APRT Deficiency Registry, housed at our institution in Reykjavik [9]. The highest cumulative minor allele frequency of confirmed pathogenic variants outside Japan and Iceland was observed in the Irish population (0.2%), which is still low. The relatively large number of affected cases in Japan and Iceland is consistent with the known founder effect in these populations [1, 2, 9]. In our aforementioned study, data collection relying primarily on published reports can be considered a limitation, as cases may have been miscounted or missed altogether [9]. The small number of reported cases suggests an exceedingly low prevalence of APRT deficiency, although limited awareness among health care professionals and missed diagnoses may certainly explain some of the discrepancies observed.

Consanguineous marriages or unions, common in certain populations, likely resulted in an underestimation of the disease prevalence in the respective geographic areas. It should, however, be noted that publicly available genomic datasets remain a limited resource that may not fully represent all populations, including certain ethnic groups. Additional work is needed to better characterize the prevalence of APRT deficiency worldwide. Nevertheless, our results do not suggest a widespread underdiagnosis of the disorder.

The Icelandic APRT Deficiency Registry is a part of the Rare Kidney Stone Consortium, an international collaborative study group dedicated to advancing the understanding, awareness, diagnosis, and treatment of rare causes of kidney stones and kidney failure. Over the last 20 years, we have (1) collected extensive high-quality data from our unique patient cohort of 63 individuals, and numerous matched urine and plasma samples, some of which have already been used in several important research projects [3, 9–13], including clinical trials [6, 14]; (2) developed laboratory assays [15, 16] for the diagnosis and therapeutic monitoring of APRT deficiency which already have significantly advanced the field of 2,8-DHA urinary stone and crystal nephropathy, both in the clinical and research settings; and (3) published other highly relevant novel information and reviews [17, 18] which significantly contribute to the scientific information presented in this educational review.

## Genetic basis of APRT deficiency

The complete nucleotide sequence of the human *APRT* was determined in 1987 [19]. The gene is located on chromosome 16q24 and is 2,466 base pairs long, comprising 5 exons that encode a 180-amino acid protein [19]. To date, we are aware of 68 pathogenic human *APRT* variants [9, 17], all of which completely abolish the enzyme function in vivo [17], and no genotype-phenotype correlations have been described [1, 2]. The 3 most commonly reported mutations, p.Met136Thr, c.400 + 2dupT, and p.Asp65Val, were found to cause 58% of cases with a molecular diagnosis in our recent study [9]. The p.Met136Thr, a missense mutation in exon 5, is the most common Japanese disease allele, affecting approximately 70–80% of patients in that population [20]. The most common European mutation, including in the French patient population, is a T insertion at the splice donor site of intron 4 (IVS4 + 2insT [c.400 + 2dupT]) [2]. A missense mutation in exon 3, p.Asp65Val, accounts for all known APRT deficiency cases in Iceland, and has also been found in Australia, Spain, France and the UK [1].

## Pathobiology of APRT deficiency

### Adenine metabolism in APRT deficiency

**Fig. 1:**
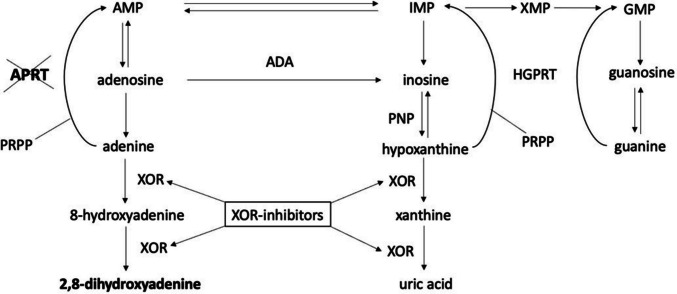
Overview of adenine metabolism in adenine phosphoribosyltransferase deficiency. When APRT is nonfunctional, adenine is oxidized by XOR to 2,8-dihydroxyadenine via 8-hydroxyadenine. Abbreviations: ADA, adenosine deaminase; AMP, adenosine monophosphate; APRT, adenine phosphoribosyltransferase; GMP, guanosine monophosphate; HPRT, hypoxanthine phosphoribosyltransferase; IMP, inosine monophosphate; PNP, purine nucleoside phosphorylase; PRPP, 5-phosphoribosyl-1-pyrophosphate; XMP, xanthine monophosphate; XOR, xanthine oxidoreductase

**Fig. 2:**
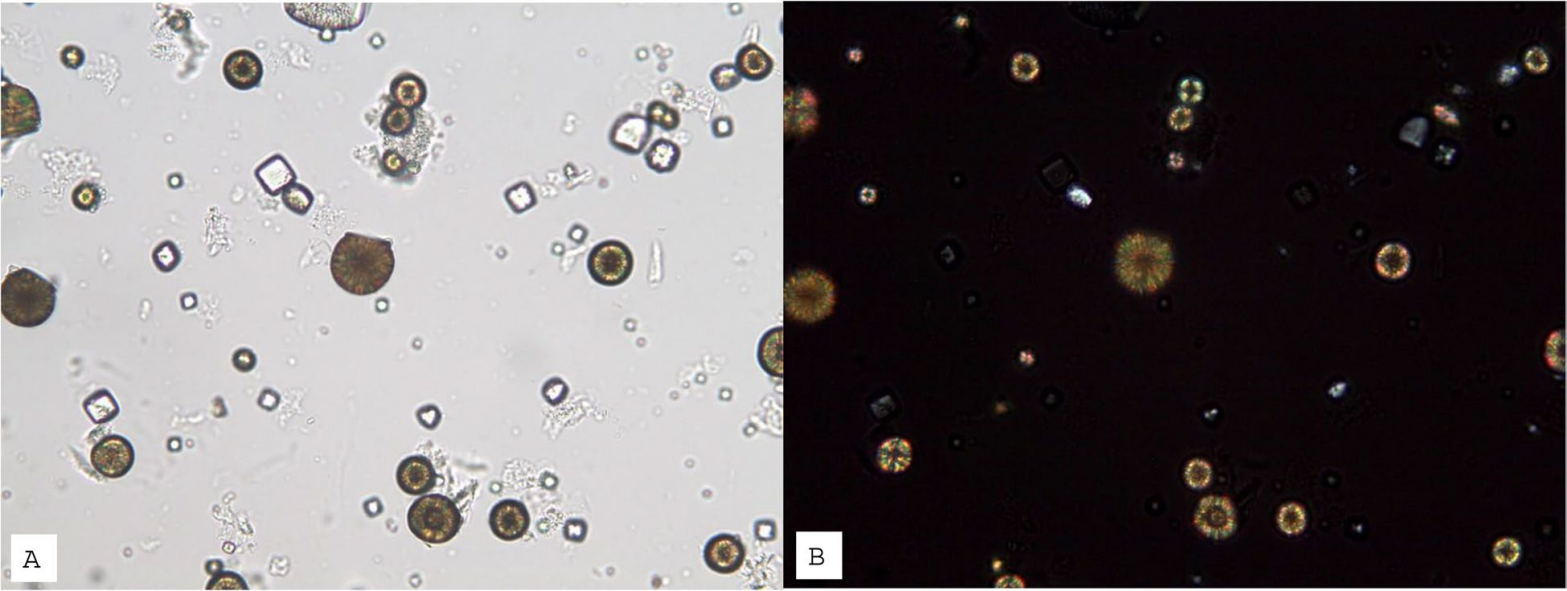
Urinary 2,8-dihydroxyadenine crystals from an individual with adenine phosphoribosyltransferase deficiency. These crystals have a characteristic appearance and polarization pattern. **A** Conventional light microscopy shows the typical brown 2,8-dihydroxyadenine crystals. Note the dark outline and central spicules (original magnification × 400). **B** The same field viewed with polarized light microscopy. The smaller crystals exhibit a central Maltese cross pattern (original magnification × 400)

A schematic overview of adenine metabolism is depicted in Fig. 1. In the human body, adenine is either synthesized de novo or recycled by APRT through the salvage pathway [18]. APRT is constitutively expressed in all tissues [21]. In healthy individuals, APRT catalyzes the conversion of adenine to 5´-adenosine monophosphate, leading to effective recycling of adenine [21]. APRT activity, measured in red cell lysates, ranges from 16 to 32 nmol/h per mg hemoglobin [17]. In persons with APRT deficiency, adenine is oxidized by xanthine oxidoreductase (XOR; previously xanthine dehydrogenase/oxidase) to the poorly soluble and nephrotoxic metabolite 2,8-DHA (Fig. 2), which is excreted in the urine in vast amounts causing stone formation and crystal-mediated kidney injury [17]. In heterozygotes and healthy individuals with the wild-type genotype, 2,8-DHA is neither detected in urine nor plasma under physiological conditions [12]. Although the level of enzyme activity in heterozygotes has been reported to be in the range of 21 to 37% of normal, rather than the expected 50% [4], these individuals remain asymptomatic throughout their whole lifespan [1]. Only a small fraction of total body adenine is of dietary origin [22], which may have implications for the limited role of purine-restricted diet in the treatment of APRT deficiency.

### Pathological features

**Fig. 3:**
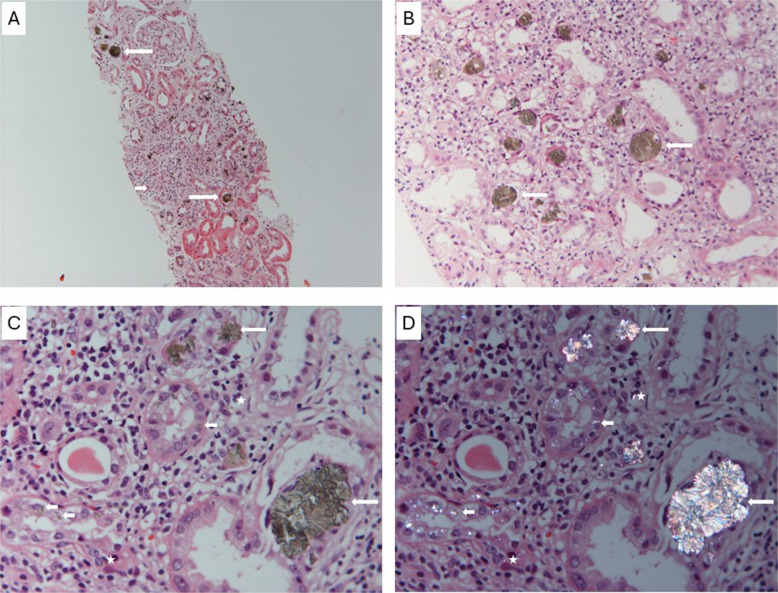
A photomicrograph of a kidney biopsy specimen from a patient with adenine phosphoribosyltransferase deficiency. **A** 2,8-Dihydroxyadenine crystals are seen within tubular lumina (arrows). Significant tubular atrophy and interstitial inflammation and fibrosis (short arrow) are present (hematoxylin and eosin stain; original magnification × 100). **B** A higher magnification of the biopsy specimen shown in **A**. 2,8-Dihydroxyadenine crystals are seen within tubular lumina (arrows) (hematoxylin and eosin stain; original magnification × 400). **C** The same biopsy specimen as shown in **A** and **B** under higher magnification. 2,8-Dihydroxyadenine crystals are observed within the tubular lumina (long arrows), inside tubular epithelial cells (short arrows) and in the interstitium (asterix) (hematoxylin and eosin stain; original magnification × 1000). **D** The same microscopic field of the kidney biopsy specimen as shown in **C** viewed with polarized light microscopy. 2,8-Dihydroxyadenine crystals are seen within tubular lumina (long arrows), inside tubular epithelial cells (short arrows) and in the interstitium (asterix) (hematoxylin and eosin stain; original magnification × 1000)

The kidney histopathology pattern observed in individuals with APRT deficiency is a crystal-associated tubulointerstitial nephropathy, found in both native and transplanted kidneys (Fig. 3) [2, 10, 17, 23]. Extensive 2,8-DHA crystal deposits are observed within tubular lumina, in the cytoplasm of tubular epithelial cells, and in the interstitial space, associated with inflammation and a variable degree of fibrosis. Interestingly, 2,8-DHA crystal nephropathy resulting in advanced CKD is not infrequently observed in individuals without a personal history of urinary stone disease [3, 23, 24]. The birefringent 2,8-DHA crystals are readily identified in the kidney parenchyma when examined under polarized light (Fig. 3) [17]. It is important not to confuse the 2,8-DHA crystal-induced kidney damage with other types of crystal-associated kidney injury, particularly oxalate-mediated crystal nephropathy [23], not the least because the currently available and effective treatment strategies vary significantly between the two disorders [14, 25]. Studies in *APRT* knockout mice have shown DHA crystal deposition and stones associated with severe kidney damage, including tubular dilation, necrosis, and interstitial inflammation and fibrosis, leading to progressive CKD and kidney failure, indistinguishable from that identified in humans with the disorder [26–29].

### Mechanism of crystal-induced kidney injury

Traditionally, crystal deposition in the kidney has been assumed to cause direct mechanical obstruction and nephron destruction, resulting in progressive CKD [30]. Although this may be true, recent studies provide evidence that inflammatory processes also mediate human 2,8-DHA crystal-induced kidney injury, including NLPR3 inflammasome activation [31–33]. While the precise mechanisms underlying 2,8-DHA crystal-mediated inflammation and fibrosis are not yet defined, some mediators appear to be critical in animal models of both oxalate and 2,8-DHA crystal nephropathies [13, 31]. Experimental studies have shown expression of genes associated with inflammation, chemotaxis and fibrosis in the kidneys of affected mice [26–29]. Our group, in collaboration with investigators from Aachen, Germany, recently demonstrated that deletion of *Tnfr1* (encoding TNF receptor 1) in a mouse model of 2,8-DHA crystal nephropathy significantly reduced tubular CD44 and annexin II expression, blunted inflammation, and ameliorated disease expression and progression [13]. In contrast, genetic deletion of *Tnfr2*, *Cd44*, or *Ahsg* had no effect on the manifestations of 2,8-DHA nephropathy.

## Clinical characteristics

Radiolucent nephrolithiasis is the most common clinical manifestation of APRT deficiency, followed by progressive CKD, AKI, and kidney failure [1, 3]. Some affected individuals remain asymptomatic into adulthood, and APRT deficiency is not infrequently diagnosed in asymptomatic individuals when 2,8-DHA crystals are incidentally detected on routine urine microscopy or when siblings of affected individuals undergo screening [1, 34]. APRT deficiency is not known to affect organs other than the kidney and urinary tract; however, the authors [6, 17] and other investigators [35] have encountered several individuals with APRT deficiency complaining of eye discomfort, although only in patients receiving XOR inhibitor (allopurinol or febuxostat) treatment [6]. Hence, the clinical manifestations are variable, and factors that may influence the phenotype are not entirely understood.

Interestingly, 15 (65%) of the first 23 Icelandic patients reported had red hair, while this was the case in only four of their 27 (15%) unaffected siblings [1]. The *MC1R* gene, which determines skin and hair pigmentation, is located close to the *APRT* gene on chromosome 16q24.3 , suggesting that the proximity of these 2 loci may influence their simultaneous transmission between generations [36].

### Childhood disease manifestations

Two studies have focused on the clinical characteristics and outcomes of APRT deficiency in children, one carried out in Iceland and the other in France [11, 34]. Urinary stone disease was the most common presentation in these two childhood studies, observed in approximately 50% of cases in the Icelandic study and 80% in the French study. A few individuals had AKI as the presenting feature. Importantly, AKI associated with bilateral urinary tract obstruction from 2,8-DHA calculi is well-documented in children [1, 37–39]. In the Icelandic study, no affected child had reached CKD category G3 or higher at the time of presentation. However, at diagnosis, which was significantly delayed in most cases, 4 of 21 individuals had progressed to CKD category G3 or higher, including one who had initiated kidney replacement therapy for kidney failure [11]. CKD with reduced kidney function was not a presenting feature in the French pediatric cohort [34]. Asymptomatic crystalluria was the presentation in 3 (14%) cases in each of the two aforementioned pediatric studies [11, 34]. Recurrent urinary tract infections, hematuria and brownish-red diaper stains are also well-known clinical manifestations of APRT deficiency presenting in childhood [1–3, 11, 34].

### Disease manifestations in adults

We have published our analyses of extensive longitudinal observational registry data from the first 53 individuals in our registry, 30 (57%) of whom were female [3]. The median age at diagnosis was 37.0 (range, 0.6–67.9) years, and after a median follow-up time of 10.3 (range, 0.0–31.5) years, 62% of patients had experienced urinary stones, 34% had developed an episode of AKI, and 42% had CKD category G3 or higher [3]. Furthermore, approximately 15% of patients had already progressed to kidney failure at diagnosis [2, 3]. We found both progressive CKD and AKI to be significant manifestations of APRT deficiency, whereas urinary stone disease is the most common presenting feature. Urinary stone disease is also the most common clinical manifestation reported by other investigators, and 60 to 90% of reported cases have already developed stones at diagnosis [1–3, 34]. Most untreated patients will experience recurrent radiolucent urinary stones, many of whom have multiple repeated clinical stone events [11]. The large proportion of patients in our study who had experienced AKI at the time of diagnosis was a novel finding [3]. The causes of AKI included biopsy-proven crystal nephropathy, obstructing urinary tract stones, and volume depletion. Interestingly, over 50% of these individuals later developed CKD category G3 or higher at a median of less than 2 years following their AKI episode [3]. We also found advanced CKD, without a history of urinary stones, to be more prevalent than previously reported. Our data also suggest that timely drug therapy significantly slows the rate of CKD progression [3]. 2,8-DHA crystal nephropathy frequently recurs in the kidney allograft and may lead to allograft loss in patients who are not adequately treated [10]. Unfortunately, the diagnosis of APRT deficiency is frequently not made until after kidney transplantation in untreated patients, when in most cases the disease has caused severe acute allograft dysfunction, or even loss of the transplanted kidney [10, 24].

## Diagnosis of APRT deficiency

Effective medical treatment is widely available for APRT deficiency, and therefore, timely diagnosis and commencement of drug treatment are essential for the prevention of adverse outcomes [3, 11, 14, 34]. Unfortunately, the lack of recognition and knowledge of APRT deficiency has frequently resulted in unacceptable diagnostic delay, often with severe consequences, including recurrent urinary stones, progressive CKD, and even recurrence of 2,8-DHA crystal nephropathy in the allograft following kidney transplantation [2, 3, 10, 11, 24]. The diagnosis of APRT deficiency should be considered in all children presenting with renal colic, radiolucent urinary stones, or AKI, and in infants with a history of reddish-brown diaper stain [1, 18]. Moreover, the diagnosis should also be contemplated in any young or middle-aged person with renal colic, radiolucent urinary stones, and progressive CKD, in particular when caused by tubulointerstitial nephropathy of unknown cause [1]. APRT deficiency should always be considered in children and teenagers with presumed uric acid urinary stones, as uric acid stones are exceedingly rare in childhood [18]. High urine pH in individuals with radiolucent stones is an additional clue to the diagnosis of APRT deficiency, as uric acid stones only develop in acidic urine.

### Urine microscopy

The characteristic 2,8-DHA crystals, which are round, reddish-brown with dark outlines and central spicules, can almost always be detected by conventional light microscopy of the urine sediment (Fig. 2A) [1]. When viewed using polarized light microscopy, the small and medium-sized 2,8-DHA crystals show the characteristic central Maltese cross pattern, which is highly suggestive of the disorder (Fig. 2B). The larger and thicker urine crystals do not always exhibit the Maltese cross pattern in polarized light, as they may be impermeable to light. The crystals, which are usually abundant in urine samples from nonoliguric individuals with APRT deficiency in the absence of XOR inhibitor therapy, are highly suggestive of the disease. However, the crystals may be scarce in urine specimens from patients with advanced CKD or kidney failure, likely due to decreased 2,8-DHA crystal clearance [1].

### Imaging

As mentioned above, 2,8-DHA urinary stones are radiolucent and will not be detected on plain abdominal radiographs [1]. The diagnosis of urinary stone disease, therefore, requires the use of imaging techniques that detect radiolucent stones, such as ultrasound (US) and/or computed tomography (CT) [18].

### Kidney stone analysis

Fourier transform infrared (FTIR) spectroscopy is currently the most widely used method for analyzing kidney stone composition [40, 41]. However, as this technique often relies on automated interpretation of FTIR spectra, it is subject to errors, and several cases of false-positive identification of 2,8-DHA as a kidney stone component have been reported by our group and others [40, 41]. Kidney stone analysis can therefore only be considered suggestive of APRT deficiency and 2,8-dihydroxyadeninura, requiring confirmatory testing in all cases.

### Kidney biopsy

The histological features of 2,8-DHA crystal nephropathy are displayed in Fig. 3. Care must be taken so that histopathologic manifestations of 2,8-DHA crystal nephropathy are not confused with those of other types of crystal-induced kidney injury, particularly oxalate- and uric acid nephropathies [23]. 2,8-DHA crystal aggregates can be mistaken for oxalate crystals, as both types of crystals are birefringent and deposit in tubular lumina, tubular cell cytoplasm, and the renal interstitium [23, 42]. Although the pathological findings are usually highly suggestive of APRT deficiency, verification is required to differentiate the disorder from other types of crystal nephropathies.

### Confirmatory diagnostic testing

The absence of APRT activity in red blood cell lysates is the original gold-standard test used to establish the diagnosis of APRT deficiency [1, 21]. Today, molecular genetic testing using targeted multi-gene panels or single-gene testing is commonly carried out to confirm the diagnosis, demonstrating biallelic pathogenic *APRT* mutations [18]. However, enzyme activity measurements should be carried out in individuals who are homozygotes or compound heterozygotes for *APRT* variants of unknown significance. 2,8-DHA is not detected under physiological conditions in human biofluids in heterozygotes or healthy persons [12]. Therefore, the demonstration of 2,8-DHA in urine or plasma, using the highly sensitive and specific ultra-performance liquid chromatography-tandem mass spectrometry (UPLC-MS/MS)-based quantification assays recently developed by our group [15, 16], can also be considered diagnostic of APRT deficiency.

### Screening for APRT deficiency

As alluded to previously, individuals with APRT deficiency are frequently asymptomatic at the time of diagnosis. In our first published study, 5 of 19 patients had no symptoms, 3 of whom were incidentally diagnosed during a routine urinalysis in our laboratory, while the other 2 were discovered through family screening [1]. As siblings have a 25% likelihood of being homozygous with no APRT activity and a 50% likelihood of being heterozygous with reduced enzyme function [1, 21], screening all siblings of known cases should be considered mandatory, including those who appear asymptomatic. Screening can be performed using genetic testing or APRT activity measurement as previously described, or by measuring 2,8-DHA in urine and/or plasma using the UPLC-MS/MS assays developed by our group [15, 16]. Skilled urine microscopy may also reveal the characteristic 2,8-DHA crystals as noted above [1]. Newborn screening may be feasible and should be considered in Iceland as well as in other populations with a high carrier frequency.

## Management of APRT deficiency

### Pharmacotherapy

Timely institution of XOR inhibitor therapy, either allopurinol or febuxostat, has in several studies, based on different levels of evidence, been found to be highly effective and safe in APRT deficiency [2, 11, 14, 34]. Published clinical trials and observational studies on the safety and effectiveness of pharmacotherapy are reviewed below.

1. Clinical trials

We recently completed a single-center, open-label, randomized, crossover clinical trial comparing the effects of allopurinol 400 and 800 mg/day and febuxostat 40 and 80 mg/day on plasma concentrations and urinary excretion of 2,8-DHA in individuals 18 years and older [6]. Plasma and urine 2,8-DHA were quantified using our UPLC-MS/MS assays [15, 16]. The plasma concentration and urinary excretion of 2,8-DHA decreased markedly with both study drugs, although febuxostat was more efficacious than allopurinol in both prescribed doses; however, it is not clear if these differences are clinically significant. A similar clinical trial was conducted by our group several years ago, comparing the effect of allopurinol 400 mg/day and febuxostat 80 mg/day on urinary 2,8-DHA excretion in patients with APRT deficiency [14]. The urinary 2,8-DHA excretion decreased markedly with both allopurinol and febuxostat treatment, and as in the more recent study, febuxostat appeared more efficacious than allopurinol in reducing DHA excretion in the doses prescribed.

2. Observational studies

We have published data on long-term kidney outcomes in 21 patients in our APRT Deficiency Registry who presented with the disorder before the age of 18 years [11]. Of these 21 patients, 14 were started on allopurinol at an age of 2.6 (range, 0.6–16.5) years, while 6 patients first began allopurinol treatment as young adults. During 18.9 (range, 1.7–31.5) years of allopurinol treatment, only 2 patients in the group treated in childhood experienced stones, and AKI occurred in 3 individuals during follow-up. In the group initiating treatment as adults, 5 patients experienced a total of 28 stone episodes, and AKI had occurred in two. At latest follow-up, estimated glomerular filtration rate (eGFR) was 114 (70–163) and 62 (10–103) mL/min/1.73 m^2^ in those who started treatment as children and adults, respectively. All 3 patients with CKD category G3 or higher at the time of last follow-up were adults when pharmacotherapy was commenced. Thus, our data show that timely diagnosis and treatment of APRT deficiency clearly ameliorates kidney complications and preserves long-term kidney function. 2,8-DHA crystal nephropathy frequently recurs in the kidney allograft and may lead to graft loss in patients who are not adequately treated [10].

The outcome of kidney transplantation in individuals with APRT deficiency is similar to that observed in patients with other causes of kidney failure when allopurinol or febuxostat therapy is initiated before the transplant procedure [24]. Data from our APRT Deficiency Registry found 2-year allograft survival to be 91% in the group treated pre-transplant compared with 55% in individuals not receiving XOR inhibitor therapy before transplantation. In that same study, the median eGFR at 2 years post-transplant was 61.3 (24.0–90.0) mL/min/1.73 m^2^ in treated individuals compared with 16.2 (10.0–39.0) mL/min/1.73 m^2^ in those without treatment (P = 0.009) [24]. In general, individuals receiving higher doses of XOR inhibitor (mostly allopurinol) seemed less likely to experience disease recurrence in the allograft. In our experience, allopurinol or febuxostat should be started at least 4 weeks prior to a kidney transplant procedure in a dose of at least 400 mg/day or 80 mg/day, respectively, regardless of the level of kidney function.

As we have encountered several individuals who had to discontinue allopurinol or febuxostat due to adverse effects [3, 6], additional treatment approaches, perhaps targeting downstream effects of 2,8-DHA and/or 2,8-DHA crystal toxicity, are needed. Furthermore, the lack of therapeutic options for the prevention of crystal-induced kidney injury, inflammation and the associated fibrosis needs to be addressed.

Other potential pharmacological treatment options, such as alkali therapy aimed at raising the pH, are not recommended for the treatment of the disorder as 2,8-DHA is highly insoluble at any physiological urine pH.

### Surgical interventions

Extracorporeal shockwave lithotripsy is an effective treatment for 2,8-DHA stones [3, 43]. The stones break easily and can be removed without much stone debris left behind. Open surgery is reserved for stones not amenable to the less invasive procedures.

### Dietary interventions

The value of a purine-restricted diet in individuals with APRT deficiency is not well studied. However, low or undetectable 2,8-DHA blood levels and urinary excretion in patients on adequate allopurinol or febuxostat treatment suggest that dietary restriction may not be of any additional value in patients receiving XOR inhibitor therapy [6, 14, 16].

## Summary and future directions

APRT deficiency is a rare but easily treatable purine metabolic disorder, leading to recurrent urinary stone formation, progressive CKD, and not infrequently kidney failure in untreated patients. Lack of familiarity with APRT deficiency among clinicians and pathologists is of concern and undoubtedly contributes to the significant diagnostic delay frequently observed, and the high proportion of patients with advanced CKD or kidney failure at the time of diagnosis. The diagnosis can be confirmed by APRT activity measurement or genetic testing which are indicated in all individuals suspected of APRT deficiency. Improving the diagnosis of patients with urinary stone disease and CKD is necessary, and the use of modern techniques such as genetic testing are increasingly uncovering rare causes of kidney stones and kidney failure, including APRT deficiency. Timely institution of targeted pharmacotherapy using allopurinol or febuxostat significantly reduces kidney stone recurrence, slows CKD progression, and likely prevents both the development of kidney failure and disease recurrence in the kidney allograft. The results of our clinical trials suggest that febuxostat more effectively reduces plasma concentration and urinary 2,8-DHA excretion than allopurinol in patients with APRT deficiency. Whether these differences are clinically significant remains to be seen. Future studies must focus on the identification of alternative drug treatments as some affected individuals do not tolerate currently available XOR inhibitor therapy. A novel therapeutic strategy based on RNA interference has been introduced into clinical practice in recent years, reducing activity of genes predominantly expressed in the liver, resulting in the mitigation of diseases such as primary hyperoxaluria [44]. Interestingly small interfering RNAs silencing the XOR enzyme are currently being tested in humans (NCT05565768) [45] and in mouse models [46] for the treatment of hyperuricemia and gout. This approach holds promise for the treatment of APRT deficiency. Furthermore, the advent of CRISPR gene editing technologies has transformed our ability to modify the genome, providing the potential to cure rare genetic diseases [47]. In APRT deficiency, effective delivery of CRISPR components may be difficult given the ubiquitous expression of the *APRT* gene.

## Key summary points

1. Although a very rare condition, APRT deficiency is an important treatable cause of recurrent urinary tract stones and progressive CKD.
2. APRT deficiency in children may present with urinary stones, AKI and in infants as reddish-brown diaper stain.
3. Genetic testing, using targeted multi-gene panels or single-gene testing, and/or APRT activity measurements are diagnostic of APRT deficiency.
4. Kidney stone analysis can be false positive and cannot be used to confirm the diagnosis of APRT deficiency.
5. Treatment with an XOR inhibitor, allopurinol or febuxostat, is highly effective and safe in APRT deficiency.
6. Kidney transplantation outcome in APRT deficiency is excellent in patients receiving appropriate XOR inhibitor treatment before surgery.

## Multiple choice questions

Answers are provided following the references.

1. Which of the following statements is true?
  - Significant genotype-phenotype correlations have been described in individuals with APRT deficiency.
  - The estimated *APRT* heterozygote frequency rate in different populations around the world is in the range of 10 to 15%.
  - The highest cumulative minor allele frequency of confirmed pathogenic *APRT* variants outside of Japan, France and Iceland has been observed in the US and Chinese populations.
  - The 3 most reported *APRT *gene mutations are p.Met136Thr, c.400 + 2dupT and p.Asp65Val and the majority of reported cases are from Japan, France, and Iceland.
  - Over 500 pathogenic human *APRT* variants have been identified.
2. Which of the following statements is true?
  - APRT activity in red blood cell lysates of less than 50% is diagnostic of APRT deficiency.
  - Individuals with severe 2,8-DHA crystal nephropathy and advanced CKD always have a personal history of urinary stone disease.
  - 2,8-Dihydroxyadenine crystal nephropathy is easily differentiated from oxalate crystal-associated kidney injury.
  - Oligoarthritis and various eye complaints are common extrarenal manifestations, reported in over 20% of individuals with APRT deficiency.
  - In APRT deficiency, adenine is oxidized by XOR to the poorly soluble and nephrotoxic metabolite 2,8-DHA which is excreted in the urine in vast amounts.
3. Which of the following statements is true?
  - Urinary stone disease is rarely identified in young (< 5 years of age) children with APRT deficiency.
  - Radiolucent kidney stones are the most common clinical manifestation of APRT deficiency, followed by CKD, kidney failure and AKI.
  - CKD category G3 and higher is a common feature of APRT deficiency in childhood.
  - Asymptomatic patients with APRT deficiency have only rarely been reported.
  - Heterozygotes frequently develop 2,8-DHA urinary stones.
4. Which of the following statements is true?
  - Molecular genetic testing using targeted multi-gene panels or single-gene testing should be carried out in all individuals suspected of APRT deficiency as the finding of biallelic pathogenic *APRT* mutations is diagnostic of the disorder.
  - Kidney stone analysis, emplyoing Fourier transform infrared (FTIR) spectroscopy, has been shown to be highly accurate for detection of 2,8-DHA stones.
  - Red hair color rules out the diagnosis of APRT deficiency.
  - The kidney diseaseobserved in individuals with APRT deficiency is 2,8-DHA crystal nephropathy, only found in native kidneys.
  - 2,8-DHA is frequently identified in the urine and plasma of heterozygotes, using highly sensitive and specific UPLC-MS/MS assays.
5. Which of the following statements is true?
  - Dietary purine restriction has been shown to be of significant value in the management of individuals with APRT deficiency, even in those receiving allopurinol or febuxostat treatment.
  - Ample fluid intake and urine alkalinization to a pH of approximately 7 should be prescribed for all individuals with APRT deficiency, including those who are already receiving allopurinol and febuxostat pharmacotherapy.
  - Recurrence of 2,8-DHA nephropathy in the transplanted kidney is usually a late event which only rarely affects one-year kidney allograft outcome (survival and function).
  - Observational cohort studies have shown that early (childhood) diagnosis of APRT deficiency and prompt institution of allopurinol or febuxostat treatment greately improves kidney outcomes by preventing new stone formation and significantly slowing the rate of or even halting CKD progression.
  - Extracorporeal shockwave lithotripsy is not an effective surgical intervention for 2,8-DHA stones.

## Supplementary Information

Below is the link to the electronic supplementary material.

Graphical abstract (PPTX 464 KB)
